# Experimental composite containing silicon dioxide-coated silver nanoparticles for orthodontic bonding: Antimicrobial activity and shear bond strength

**DOI:** 10.1590/2177-6709.27.3.e222116.oar

**Published:** 2022-07-04

**Authors:** Rogéria Christina de Oliveira AGUIAR, Larissa Pereira NUNES, Eduardo Silva BATISTA, Marina Mariante VIANA, Marcela Charantola RODRIGUES, Bruno BUENO-SILVA, Marina Guimarães ROSCOE

**Affiliations:** 1 Universidade de Guarulhos (UNG), Faculdade de Odontologia (Guarulhos/SP, Brazil).; 2 Universidade Estadual de São Paulo (UNESP), Departamento de Medicina Dentária Preventiva e Restaurativa, Faculdade de Odontologia (Araçatuba/SP, Brazil).; 3 Universidade Cruzeiro do Sul (UNICSUL), Departamento de Pós-graduação, Faculdade de Odontologia (São Paulo/SP, Brazil).; 4 Universidade Municipal de São Caetano do Sul, Programa de Pós-Graduação em Ensino em Saúde (São Caetano do Sul/SP, Brazil).; 5 Universidade de São Paulo (FOUSP), Departamento de Biomateriais e Biologia Oral, Faculdade de Odontologia (São Paulo/SP, Brazil).

**Keywords:** Dental materials, Orthodontics, Composites resins, Demineralization, Nanoparticles

## Abstract

**Objective:**

This study aimed to investigate the antimicrobial activity and shear bond strength (SBS) of orthodontic brackets to bovine enamel using experimental composites with different concentrations of silicon dioxide-coated silver nanoparticles (Ag@SiO_2_ NPs).

**Methods:**

Fifty bovine incisors were divided into five groups according to the composite (n = 10): G1 - Control Group (Transbond XT Resin), G2 - Experimental composite without Ag@SiO_2_ NPs; G3 - Experimental composite with 0.5% of Ag@SiO_2_ NPs; G4 - Experimental composite with 1% of Ag@SiO_2_ NPs; G5 - Experimental composite with 3% of Ag@SiO_2_ NPs. The SBS test was performed using a universal mechanical testing machine, and the adhesive remnant index (ARI) was analyzed by optical microscopy. For the antimicrobial activity evaluation, *Streptococcus mutans* (*S. mutans*) biofilm was formed for three days in hydroxyapatite discs. Posteriorly, *S. mutans* colony forming units (CFU) were evaluated. For SBS analysis, Analysis of Variance was used, followed by the Tukey test, at a 5% statistical significance level. The CFU data were analyzed by Kruskal-Wallis, followed by Dunn as a *post-hoc* test. The ARI results were analyzed descriptively.

**Results:**

There was no statistically significant difference in SBS values between the experimental and control groups (*p*>0.05). A 3% incorporation of Ag@SiO_2_ NPs statistically reduced the SBS values (*p*<0.05) compared to the 1% group. The addition of 3% of Ag@SiO_2_ NPs to the composites significantly reduced *S. mutans* biofilm formation, compared to group G2 (*p*<0.05).

**Conclusion:**

Composites incorporating 3% of Ag@SiO_2_ NPs presented similar SBS values compared to the control group, and showed significant antimicrobial activity.

## RESUMO

**Objetivo:**

O presente estudo objetivou investigar a atividade antimicrobiana e a resistência de união ao cisalhamento (RU) de braquetes ortodônticos colados em esmalte bovino, utilizando compósitos experimentais com diferentes concentrações de nanopartículas de prata revestidas com dióxido de silício (NPs Ag@SiO_2_).

**Material e Métodos:**

Cinquenta incisivos bovinos foram divididos em cinco grupos, de acordo com o compósito utilizado (n = 10): G1 - Grupo Controle (Resina Transbond XT); G2 - Compósito Experimental sem NPs Ag@SiO_2_; G3 - Compósito Experimental com 0,5% de NPs Ag@SiO_2_; G4 - Composto Experimental com 1% de NPs Ag@SiO_2_; e G5 - Compósito Experimental com 3% de NPs Ag@SiO_2_. O teste de RU foi realizado em máquina universal de ensaios mecânicos, e o índice de adesivo remanescente (IAR) foi analisado por microscopia óptica. Para a avaliação da atividade antimicrobiana, biofilme de *S. mutans* foi formado por três dias em discos de hidroxiapatita. Posteriormente, foram avaliadas as unidades formadoras de colônias (UFC) de *S. mutans*. Para a análise de RU, foi utilizada a Análise de Variância, seguida do teste de Tukey, com nível de significância estatística de 5%. Os dados de UFC foram analisados por meio do teste de Kruskal-Wallis, seguido do teste *post-hoc* de Dunn. Os resultados de IAR foram analisados descritivamente.

**Resultados:**

Não houve diferença estatisticamente significativa nos valores de RU entre os grupos experimentais e o grupo controle (*p*> 0,05). A incorporação de 3% de NPs Ag@SiO_2_ reduziu estatisticamente os valores de RU (*p*< 0,05), em comparação ao grupo com 1%. A adição de 3% de NPs Ag@SiO_2_ ao compósito reduziu significativamente a formação de biofilme de *S. mutans*, em relação ao grupo G2 (*p*< 0,05).

**Conclusão:**

Os compósitos com incorporação de 3% de NPs Ag@SiO_2_ apresentaram valores de RU semelhantes ao grupo controle e demonstraram significativa atividade antimicrobiana.

## INTRODUCTION

White spot lesions (WSLs) are a great concern for orthodontists. The prevalence of WSLs, based on post-treatment evaluations, ranged from 25 to 97%.^1^ ^-^ ^3^ This high frequency is related to the fact that orthodontic accessories increase the risk of biofilm retention (especially *Streptococcus mutans, S. mutans*), create numerous retention sites, and hamper adequate oral hygiene.^4^ ^-^ ^6^ The incorporation of antimicrobial agents into dental materials has been pointed out as an effective strategy to reduce WSLs risk, since there is no need of patient compliance for effective biofilm control.^7^

The ideal antimicrobial agent should present a broad-spectrum action,^8^ ^,^ ^9^ long-term duration,^10^ ^,^ ^11^ and no toxicity.^12^ Furthermore, its incorporation should not negatively interfere with optical and mechanical properties.^13^ ^,^ ^14^ Silver particles present most of these desirable characteristics, such as a broad spectrum of action and low antimicrobial resistance.^15^ ^-^ ^19^ Still, its addition causes significant material color changes,^7^ compromising aesthetics, which is not acceptable, especially for patients with ceramic appliances. Therefore, recently, studies have proposed the synthesis of silicon dioxide-coated silver nanoparticles as an alternative to masking the material metallic coloration.^20^

The antimicrobial efficacy of silver nanoparticles against the main caries pathogens, especially *S. mutans*, is well known.^21^ ^-^ ^23^ Recently, studies have shown that silver nanoparticles (50 nm) addition to commercial composites reduced up to 40% of the *S. mutans* population.^22^ When smaller particles (20 nm) were added, the results were even more expressive, providing a 94% reduction of *S. mutans.* ^23^ It is important to emphasize that, although some studies have shown that silver nanoparticles can have a cytotoxic potential and some side effects in the surrounding dental tissues, these harmful effects are depended on factors such as concentration and particle size used.^24^ ^,^ ^25^ For the composites’ synthesis, only a low concentration of silicon dioxide-coated silver nanoparticles is necessary, which does not present any cytotoxic potential.^20^

Based on the fact that fixed orthodontic treatment carries a high risk of WSLs development, it seems promising to investigate the effect of incorporating different concentrations of silica-coated silver nanoparticles (Ag@SiO_2_ NPs) on the mechanical property and antimicrobial activity of experimental composites. The null study hypothesis was that the addition of Ag@SiO_2_ NPs (0.5%, 1%, and 3%) would not reduce the *S. mutans* population around orthodontic brackets and would not affect its shear bond strength to the enamel.

## MATERIAL AND METHODS

### MANIPULATION OF THE EXPERIMENTAL COMPOSITES

The organic matrix was prepared with BisGMA (2.2bis [4-(2-hydroxy-3-metacryloxpropoxy) - propane, ESSTECH Technology Inc., Essington, PA) and TEGDMA (2-methyl 2-propoic acid, ESSTECH) at 1:1 (mol). Photoinitiators DMAEMA (Sigma-Aldrich Inc., USA) and camphorquinone (Sigma-Aldrich Inc., Germany) were added at 0.5 wt%. Barium glass was added at 70 wt% in the experimental composite without Ag@SiO_2_ NPs, to achieve a similar filler content of the commercial material (Transbond XT, 3M ESPE, USA). The silver nanoparticles coated with silicon dioxide were synthesized and characterized according to Rodrigues et al*,* ^20^ and added to this polymeric matrix at concentrations of 0.5%, 1%, and 3% by weight. The barium glass content was adjusted accordingly, to obtain 70 wt% of the filler content for all groups. As a comparison standard, a commercial composite was also tested (Transbond XT, 3M ESPE, USA).

### BRACKET-TOOTH SHEAR BOND STRENGTH (SBS)

Fifty freshly-extracted bovine incisors were randomly divided into five groups (n = 10). Before the adhesive procedure, prophylaxis was performed using rubber cups and pumice paste. Teeth surfaces were etched with 37% phosphoric acid (Dentsply, USA) for 30 seconds in the center of the buccal surface, in a standardized area corresponding to the size of the base of the metallic brackets of the maxillary incisors (Morelli, Brazil). Then, the teeth were washed with water spray for twice the etching time, and the enamel was air-dried. For the commercial group (G1), a thin layer of the orthodontic primer Transbond XT (3M ESPE, USA) was applied to the etched surface and air-dried. Then, the Transbond XT composite was applied to the metallic brackets base and light-cured for 20 seconds. For the experimental groups (G2, G3, G4, and G5), a universal adhesive (Tetric N-Bond, Ivoclar Vivadent, Liechtenstein) was applied to the etched surface as a thin layer and air-dried. The experimental composites were then applied to the base of the brackets and light-cured for 40 seconds. The brackets were pressed to the center of the buccal face, with a 500-gram force (gf) pressure standardized with a tensiometer, followed by removing composite excess using dental explorer #5 (Duflex, Brazil). All light activations were performed using a polywave light-emitting diode (Bluephase N, Ivoclar Vivadent, Liechtenstein), with an irradiance of 1200 mW/cm².

**Figure 1:**
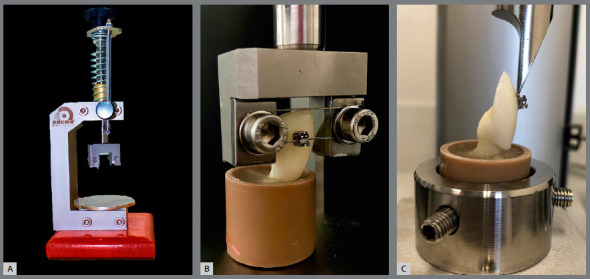
A) Parallelometer device. B) Sample included in the PVC cylinder with the aid of the parallelometer. C) Cylinder containing tooth fixed in a universal mechanical testing machine for shear bond strength testing.

To assist the specimen’s preparation for the SBS test (embedding and alignment inside the PVC pipe), a device with a system for fixing the tooth bracket into a rectangular orthodontic wire was used to guarantee perpendicularity of the specimen and evaluation of the adhesive interface (Odeme Dental Research, Brazil) (Fig 1). The specimens were then stored in distilled water at 37°C for 72 hours. 

The mechanical test was performed in a universal machine of tests (Shimadzu, Japan). Samples were positioned on a shear device with a knife-edged chisel (Odeme Dental Research, Brazil) placed at the enamel-bracket interface (Fig 1). A compressive load was applied at a 0.5-mm/min crosshead speed until failure. The results were obtained in kilogram-force (kgf), and shear bond strength values were calculated in Megapascals (MPa) considering the bracket base area. 

The debonded surfaces were examined using a stereomicroscope (ZEISS Axiocam 105 color, Germany) at 8X magnification. The amount of adhesive left on the surface was scored by using the adhesives remnant index (ARI).^26^ The ARI scale ranges from 0 to 3: 0 indicates no adhesive remaining on the tooth; 1, less than half of the enamel bonding site covered with adhesive; 2, more than half of the enamel bonding site covered with adhesive; and 3, enamel site covered entirely with adhesive.

### ANTIMICROBIAL ACTIVITY TEST

Orthodontic brackets were bonded with commercial and experimental composites, either without the incorporation of Ag@SiO_2_NP (G2) or modified by the addition of 0.5, 1, and 3 wt% of Ag@SiO_2_NPs (G3, G4, and G5, respectively) to hydroxyapatite discs (12.3-mm diameter, 1.43-mm thickness - “Clarkson Calcium Phosphates”, USA), used as the substrate for biofilm formation. The photoactivation was performed for 40 seconds using a polywave light-emitting diode (Bluephase N, Ivoclar Vivadent, Liechtenstein) with an irradiance of 1200 mW/cm^2^. 

*S. mutans* UA159 was reactivated from stock cultures in Brain Heart Infusion Media (BHI) for 18-24 hours at 37°C, 5% CO_2,_ and grown in BHI agar plates. After microbial growth, individual colonies were removed with a platinum loop and then suspended in the BHI medium to start the growth curve. After 3 hours and 30 minutes, *S. mutans* reached the mid-log phase and were used as inoculum for biofilm formation.^27^

A volume of 100 µL of log-phase microbial suspension (described above) was inoculated into 100 mL of BHI medium with 1% of sucrose, to obtain a bacterial concentration around 1-2 x 10^5^CFU/mL. Immediately after homogenization, a volume of 2.5 mL of the inoculated medium was added to each well of the 24-wells microplate with hydroxyapatite discs.

The monospecies biofilms of *S. mutans* UA 159 were formed on hydroxyapatite discs with bonded brackets for 116 hours (5 days) and incubated at 37ºC, 5% CO_2_, with daily changes of culture medium. At the end of the 5th day of biofilm formation, biofilms were sonicated, diluted, and the microbial count (colony forming units /mL count) was performed.^27^

### STATISTICAL ANALYSIS

For analysis of the shear bond strength values, one-way ANOVA was used, followed by the Tukey test, at a 5% statistical significance level. The CFU data were analyzed by Kruskal-Wallis, followed by Dunn as a *post-hoc* test. The chi-square test was used to determine significant differences in ARI scores among the groups.

## RESULTS

### BRACKET-TOOTH SHEAR BOND STRENGTH (SBS)

The results of the SBS test are displayed in Table 1. One-way ANOVA revealed a statistically significant difference in SBS among the groups (*p*< 0.05). When compared to the commercial group (G1), there was no significant difference among groups with the incorporation of Ag@SiO_2_ NPs, regardless of their concentration (*p*> 0.05). However, the 3% incorporation of Ag@SiO_2_ NPs (G5) statistically reduced the bracket-tooth shear bond strength, when compared to the group with 1% of nanoparticles incorporated (G4) (*p*< 0.05).

**Table 1:** Mean, 95% confidence interval (IL - inferior limit; SL - superior limit), standard deviation (SD), median, minimum and maximum shear bond strength values (MPa).

Mean		CI 95%		SD	Median	Minimum	Maximum
		IL	SL				
G1	29.16^AB^	24.18	34.14	6.96	31.63	12.42	38.31
G2	29.35^AB^	24.99	33.70	6.09	32.46	20.72	35.34
G3	31.55^AB^	26.92	36.17	6.47	31.52	18.68	40.60
G4	33.20^B^	30.46	35.94	3.83	31.58	28.96	39.74
G5	24.64^A^	20.18	29.10	6.24	24.96	13.85	33.10

* Distinct letters indicate presence of statistical difference between the groups (*p* < 0.05).

The frequency distribution of the ARI and the chi-square comparison of the groups are displayed in Table 2. There was no significant difference between the groups (*p*> 0.05). The bond failure pattern was similar in all the evaluated composites. The most frequent failure pattern among the groups indicated less than half of the enamel bonding site covered with adhesive (ARI 1). 

**Table 2:** Frequency distribution of the adhesive remnant index (ARI) scores.

ARI scores	G1	G2	G3	G4	G5
0	0	0	1	0	0
1	9	10	9	10	9
2	1	0	0	0	1
3	0	0	0	0	0

### ANTIMICROBIAL ACTIVITY TEST

Table 3 shows the results of the *S. mutans* biofilm formation test. The addition of Ag@SiO_2_NPs at 3% (G5) to the orthodontic experimental composite reduced the CFUs from biofilm formation compared to the experimental composite without Ag@SiO_2_ NPs (G2) (n = 6; *p*< 0.05).

**Table 3:** Counts of biofilm colony forming units (CFUs) formed on hydroxyapatite discs with brackets bonded with the different experimental groups: G1 (commercial adhesive); G2 (Experimental composite without Ag@SiO_2_ NPs); G3 (Experimental composite with 0.5% of Ag@SiO_2_ NPs); G4 (Experimental composite with 1% of Ag@SiO_2_ NPs); G5 (Experimental composite with 3% of Ag@SiO_2_ NPs).

**CFU of *S. mutans* biofilm**				
G1	G2	G3	G4	G5
2.47E+09^a,b^	2.70E+09^a^	1.89E+09^a,b^	9.83E+08^a,b^	8.46E+08^b^
(± 4.11E+08)	(± 1.42E+09)	(± 8.69E+08)	(± 3.18E+08)	(± 1.58E+08)

* Distinct letters indicate presence of statistical difference between the groups (*p* < 0.05).

## DISCUSSION

The null hypothesis tested was rejected. Composites containing 3% of Ag@SiO_2_NPs presented significant antimicrobial activity (70% reduction of CFUs), compared to the experimental group without NPs (G2). Additionally, there was a statistically significant difference in SBS values between groups with the incorporation of 1% and 3% of Ag@SiO_2_ NPs.

Although there is no consensus on the mechanism of action of silver particles, authors believe that it occurs by superficial contact with microorganisms,^28^ and that its antibacterial effect increases as smaller particles are used.^29^ In this study, Ag NPs with 11 nm were used, and a small area of the material (bracket cementation line) was exposed to the oral environment. It is essential to highlight that, although the antimicrobial effect of Ag NPs is proven in the scientific literature, there are still no scientific studies that have evaluated the impact of incorporating Ag@SiO_2_ NPs in experimental orthodontic composites.

The ability to mask the material metallic coloration is the inherent advantage of these nanoparticles, compared to the conventional silver particles.^20^ Also, the present results showed that the incorporation of 3 % of the Ag@SiO_2_ NPs into the experimental material presented a significant antimicrobial activity. The scientific literature presents positive results regarding the antimicrobial potential of materials with silver nanoparticles with concentrations ranging from 0.5 to 1% in weight.^22^ ^,^ ^23^ Moreover, a recent research demonstrated antimicrobial activity against *S. mutans* of Ag@SiO_2_ NPs not incorporated at any material.^20^ It is worth mentioning that a higher percentage of silver than the one reported in the literature was incorporated to evaluate the resin antimicrobial activity, since the effective mass of silver in nanoparticles coated with silicon dioxide is less than the same percentage of uncoated nanoparticles (18% of the nanoparticle mass percentage corresponds to silicon dioxide).

It is also necessary to investigate its influence on mechanical properties, especially when used for bonding orthodontic brackets.^30^ Ideally, the bracket should be maintained fixed to the tooth throughout the orthodontic treatment, and it should be removed without any damage to the enamel structure. ^31^ The SBS test represents the method of choice to evaluate the efficiency of orthodontic bonding systems.^32^ ^,^ ^33^ The scientific literature points out that the minimum value of SBS for clinical use varies between 5.9 and 7.8 MPa.^34^ Despite a very classic reference, this study presents important limitations. Therefore, a commercial composite control group was included for comparison (Transbond XT), since it represents the gold standard material in several studies, presenting SBS values ranging from 7 MPa up to 19 MPa.^32^ ^,^ ^35^ ^,^ ^36^ When comparing the present study results with the reference values^34^ and with previous studies,^32^ ^,^ ^35^ ^,^ ^36^ all materials presented higher resistance values, ranging from 24.64 to 33.20 MPa, indicating their suitability for clinical use.

Higher SBS values are commonly associated with a higher risk of enamel damage during debonding.^37^ Despite high SBS values, the optical microscopy analysis was unable to identify any enamel damage. The most frequent failure pattern indicated less than half of the remaining adhesive bonded to the tooth (score 1), regardless of the group evaluated. More than 90% of the samples tested presented little or almost no amount of adhesive bonded to the tooth after debonding. The advantage of detecting a few adhesive remnants bonded to the enamel is related to the lower chance of surface damage during remnant removal. Still, some authors believe that adhesive failure would ideally occur at the adhesive-bracket interface, resulting in a large amount of remaining adhesive bonded to the enamel (score 3) and a lower risk of enamel fracture.^38^ Conflicting with our results, this last scenario has been widely observed in previous studies in which the Transbond XT composite was used.^39^ ^,^ ^40^ ^,^ ^41^ Still, some authors have also reported that less than half of the composite remained in the tooth, with the highest prevalence in the Transbond XT groups,^42^ corroborating with the present study results. Similarly, according to Hellak et al*,* ^43^ the Transbond XT group showed more frequently the score 0, with no significant enamel changes after debonding. 

This manuscript represents an initial and promising *in vitro* study. More research should be performed to identify the optimal concentration that would enable antimicrobial effect with no negative influence on the orthodontic bonding material’s mechanical properties. Future clinical and *in situ* studies should also be performed to monitor aging’s impact, to verify the bonding stability and the antimicrobial activity during fully orthodontic treatment. 

## CONCLUSION

The addition of 3 wt% silicon dioxide-coated silver nanoparticles to dental adhesives reduced the *S. mutans* population around orthodontic brackets. Moreover, it did not affect its SBS to enamel, when compared to a commercial orthodontic composite resin.
